# 肺磨玻璃结节临床研究进展

**DOI:** 10.3779/j.issn.1009-3419.2016.02.08

**Published:** 2016-02-20

**Authors:** 镭 李, 丹 刘, 盈盈 朱, 为民 李

**Affiliations:** 610041 成都，四川大学华西医院呼吸内科 Department of Pulmonary and Clinical Care Medicine, West China Hospital, Sichuan University, Chengdu 610041, China

**Keywords:** 肺肿瘤, 磨玻璃结节, 影像学, 病理分型, Lung neoplasms, Ground-glass nodules, Imaging, Pathological types

## Abstract

磨玻璃结节（ground-glass nodules, GGNs）是肺结节中的特殊类型，随着高分辨薄层计算机断层扫描（high resolution computed tomography, HRCT）的应用，GGNs检出率逐年升高并受到日益广泛的关注。由于缺乏特征性临床症状，肺癌的早期诊断难度较大，而既往研究证实GGNs的出现常常提示与肺癌相关，因此加强筛查及管理有助于早期诊断及治疗肺癌。本文回顾既往相关研究，就GGNs的定义、分类、影像学特征、自然生长史、分子病理特征及诊治流程作一小结。

## Abstract (en)

Ground-glass nodules (GGNs) was a special type of pulmonary nodules. With the progress of high resolution CT (HRCT), it achieved a higher positive rate and attracted much attention in recent years. For lacking characteristic symptoms, the early diagnosis of lung cancer was difficult even nowadays. However, it had been proved that GGNs was well associated with lung cancer in previous studies. Therefore, optimized managements of GGNs could help diagnosis and treatments of lung cancer at early stage. In this review, we summarized the definition, classification, imaging characteristics, growing history, molecular pathological features and suggested managements of GGNs.

随着计算机断层扫描（computed tomography, CT），尤其是高分辨薄层CT（high resolution CT, HRCT）的广泛使用，肺部结节的检出率逐年升高。磨玻璃结节（ground-glass nodules, GGNs）为其中的特殊类型，他的出现与肿瘤密切相关，同时常常提示病变处于早期阶段。因此，加强对GGNs的认识可有效地指导随访及治疗，有助于改善肺癌患者预后。

## 1 概述

GGNs又被称为磨玻璃影（ground-glass opacities, GGOs），指肺内局灶性、结节状、淡薄密度增高影，结节内部原有结构如血管、气道及小叶间隔仍可见^[1]^。GGNs可分为两大类，其中不含实性成分的为单纯性GGNs（pure GGNs, pGGNs）；伴有实性成分、掩盖部分肺纹理的为混合性GGNs（mixed GGNs, mGGNs）或部分实性GGNs（part-solid GGNs）^[2]^。

GGNs是一种非特异性病变，其形成与含气腔内的局部浸润有关。当肺泡腔或腺泡内存在液体渗出、炎性浸润、出血或新生物时，局部组织密度增高，气体含量减少，即可出现GGNs。GGNs可为良性病变，如局灶性间质纤维化（focal interstitial fibrosis, FIF）、感染、出血、水肿等；可为腺癌浸润前病变，如原位腺癌（adenocarcinoma *in situ*, AIS）、非典型腺瘤样增生（atypical adenomatous hyperplasia, AAH）等；也可为恶性病变，如微浸润性腺癌（minimally invasive adenocarcinoma, MIA）、浸润性腺癌（invasive adenocarcinoma, IA）等^[3, 4]^。

## 2 GGNs与肺癌相关性研究

既往多项研究关注GGNs在肺癌中所占比例，但由于纳入人群基线情况的差异，目前尚无一致结论。总体而言，与实性结节相比，GGNs与肺癌的关系更为密切。尤其是mGGNs的出现，常常高度提示肺腺癌。而就肺鳞癌而言，除少数个案报道提出GGNs最终被确诊为鳞癌外^[5, 6]^，其余研究均证实GGNs与肺鳞癌无直接相关性^[7, 8]^。

Henschke等^[9]^回顾性纳入了233例低剂量CT发现的肺结节，44例（19%）为GGNs，其中15例诊断为肺癌，其恶性构成比为34.1%；而实性结节仅7%为肺癌。进一步研究发现，上述肺结节中pGGNs及mGGNs的恶性构成比分别为18%（5/28）、63%（10/16），后者远高于前者（*P* < 0.05）。该结论在后续多项研究中被证实：Cho等^[10]^对330例已接受手术治疗的GGNs患者进行研究发现，其恶性构成比为95.2%（314/330），其中AIS占12.1%（*n*=38），MIA占20.1%（*n*=63），IA占67.8%（*n*=213）；在Heo等^[11]^的研究中，90%（45/50）GGNs被诊断为肺癌。Yamaguchi等^[12]^回顾性纳入了33例手术患者、共47例pGGNs进入研究，分析发现高达41例（87.2%）结节病理诊断为腺癌。此外，在Kim等^[13]^的研究中49例pGGNs被纳入，随访1个月无变化；分析发现40例（81.6%）被诊断为肺癌，分别为细支气管肺泡癌（broncholoalveolar cell carcinoma, BAC）（*n*=36）、以BAC为主的腺癌（*n*=4）。

## 3 GGNs与肺腺癌病理分型、影像学特征的相关性研究

2011年，国际肿瘤研究协会（International Association for the Study of Lung Cancer, IASLC）、美国胸科协会（American Thoracic Society, ATS）以及欧洲呼吸学会（European Respiratory Society, ERS）对肺腺癌的分型制订了新的标准^[14]^。多项研究发现根据该分型标准，各亚型与GGNs的不同影像学表现具有明显相关性。

AAH在CT上通常表现为直径 < 5 mm的pGGNs，极少数情况下直径可达1 cm^-2^ cm^[15, 16]^。在Oda等^[17]^的研究中，单因素及多因素分析均证实圆球形pGGNs与AAH相关（OR=0.059, *P* < 0.001; OR=0.125, *P*=0.042）。非粘液性AIS一般为直径 > 5 mm的pGGNs，部分病例由于内部结构崩解也可表现为mGGNs^[18]^。粘液性AIS较为少见，多为孤立性实性结节^[14]^。手术切除后，AIS的疾病相关生存率可达100%^[13]^。

MIA是IASLC/ATS/ERS分类标准中新增的类型，目前相关研究较少。由于间质浸润深度≤5 mm，非粘液性MIA通常表现为pGGNs或含有极少量实性成分的mGGNs^[19]^。粘液性MIA更为罕见，通常表现为实性为主的结节^[14]^。在Zhang等^[20]^的研究中，MIA被证实多为较大的、分叶或边缘不规则的mGGNs，且实性成分直径多大于5 mm。MIA预后佳，手术切除后疾病相关生存也可达100%^[14]^。

实性为主型IA表现为实性结节，边缘多光滑^[21]^。贴壁状为主型IA多为实性结节或以实性为主的pGGNs，极少数可表现为mGGNs^[22]^；其亚实性成分为贴壁生长的细胞，而实性成分则为浸润性病变；多分布在外周，边缘常不清楚^[21]^。Lim等^[23]^研究发现，支气管气相（*P*=0.012）、结节直径（cutoff=16.4 mm, *P*=0.032）以及结节质量（cutoff=0.472, *P*=0.040）等因素可有效鉴别IA与AIS、MIA。其他种类的IA多表现为实性结节或以实性成分为主的mGGNs，且其预后普遍较贴壁状为主型差。

此外，既往研究多采用Noguich分型标准^[24]^进行病理分型，其病理影像学特征与上述研究结果一致。

## 4 GGNs影像学特征与肺癌的相关性研究

GGNs的发现依赖于体检筛查。既往筛查手段主要为胸部X光，但由于GGNs密度较低，胸片上常常较难发现，易发生漏诊。近年来，随着胸部CT、特别是HRCT的广泛使用，对GGNs的诊断得到了大幅提升。

### 4.1 GGNs大小与肺癌

Cho等^[10]^的研究发现，良性GGNs的直径（15.1±9.3）mm明显小于恶性GGNs（20.3±11.0）mm，较大直径是恶性病变的独立危险因素（OR=1.086; 95%CI: 1.001-1.178; *P*=0.047）。Heo等^[11]^的研究也得到了一致结论，良性与恶性GGNs的最大径分别为（11±3）mm、（19±9）mm（*P* < 0.05）。但在Fan等^[25]^的研究中，对82例GGNs进行统计分析发现：良性GGNs的最大径（23.3±7.1）mm与恶性GGNs（23.6±7.3）mm无明显差异；Lee等^[26]^纳入96例GGNs研究发现：最大径 > 10 mm的GGNs与最大径≤10 mm的GGNs恶性构成比相近（50% *vs* 51.2%）。造成不同结论的原因可能为纳入人群存在差异：前两项研究纳入标准为手术切除的GGNs，对于术前明确诊断的GGNs予以排除，其恶性构成比分别为95.2%及90.0%；而后两项研究纳入标准为CT筛查发现的GGNs，确诊方式包括手术、活检及临床诊断，恶性构成比分别为74.3%、51.0%，明显低于前者。

### 4.2 实性成分的比例与肺癌

既往研究证实：mGGNs恶性程度较pGGNs高，但关于实性成分比例与恶性程度的关系，不同研究间存在差异。在Matsuguma等^[27]^的研究中，根据实性成分比例，GGNs被分为了Ⅰ度（0%）、Ⅱ度（1%-25%）、Ⅲ度（26%-50%）、Ⅳ度（51%-75%）、Ⅴ度（76%-100%），结果表明实性成分增多是淋巴结转移的独立危险因素（OR=4.87; 95%CI: 1.51-15.77），实性成分比例越高，肺癌侵袭能力越强。Lee等^[26]^纳入18例GGNs，采用类似分度标准，将GGNs分为1度（≤25%, *n*=6）、2度（26%-50%, *n*=4）、3度（51%-75%, *n*=5）、4度（> 75%, *n*=3），其恶性构成比（83%, 100%, 100%, 100%）无明显差别。

### 4.3 GGNs边缘及内部征象与肺癌

一项纳入82例GGNs的研究发现，分叶征（14.3 *vs* 83.6, *P* < 0.001）、短毛刺征（4.8 *vs* 34.4, *P*=0.008）、长毛刺征（0.0 *vs* 29.5, *P*=0.004）、边界不清（66.7 *vs* 1.6, *P* < 0.001、不规则边缘（33.3 *vs* 93.4, *P* < 0.001）、含气征（14.3 *vs* 59.0, *P* < 0.001）、胸膜凹陷征（4.8 *vs* 70.5, *P* < 0.001）、血管集束征（4.8 *vs* 36.1, *P*=0.006）等均提示恶性病变^[25]^。Heo等^[11]^的研究也发现，在50例GGNs患者中空泡征和不规则边缘仅见于恶性GGNs。上述结论被Lee等^[28]^的研究证实：恶性pGGNs、恶性mGGNs出现分叶征的比例明显高于良性（63.6% *vs* 8.0%, *P* < 0.05; 94.4% *vs* 62.5%, *P* < 0.05），且分叶征是恶性病变的独立危险因素（OR=13.769, *P*=0.016; OR=10.200, *P*=0.024）。

但在另一项纳入53例pGGNs的研究中，Kim等^[13]^将GGNs的边缘分为光滑、毛刺、分叶及毛刺伴分叶，分析发现边缘特征与GGNs的恶性程度无明显相关性。

## 5 GGNs与PET/CT的相关性研究

正电子发射-计算机断层扫描（positron emission tomography-computed tomography, PET/CT）是鉴别肺结节良恶性的重要手段，他结合了CT及FDG-PET两种检查方式，其中前者可检出形态结构改变，而后者提供细胞生长、代谢等功能学信息。一般情况下，肿瘤细胞对氟[^18^F]脱氧葡萄糖（^18^F-fluorodeoxyglucose, ^18^F-FDG）摄取率高，在PET/CT中表现为SUV值（maximum standardized uptake value, SUVmax）增高。多项研究发现，SUV值的改变有助于鉴别肺结节良恶性。Lowe等^[29]^证实：SUV值> 2.5可诊断恶性肺结节，其敏感度、特异度分别为92%、90%。但对于GGNs而言，由于其生长代谢不活跃，PET/CT较易出现假阴性。Chun等^[30]^对68例GGNs研究发现：恶性pGGNs与良性pGGNs相比，SUV值接近[(0.64±0.19), range 0.43-0.96; (0.74±0.28), range 0.32-1.00]，差异无统计学意义（*P*=0.37）；而恶性mGGNs与良性mGGNs相比，前者SUV值[(1.26±0.71), range 0.32-2.6; (2.00±1.18), range 0.48-5.60]较小，差异有统计学意义（*P*=0.018）。Tsushima等^[31]^的研究发现：良性GGNs的平均SUV值及最大SUV值明显高于恶性GGNs（*P* < 0.001），且SUV > 1.5用于诊断良性病变的敏感度、特异度、准确度分别为100.0%、96.4%、100.0%。但Chiu等^[32]^的研究发现：最大SUV值在良恶性GGNs间无明显差异。上述研究表明PET/CT在鉴别GGNs良恶性方面并非首选。此外，GGNs通常不伴随淋巴结转移及远处转移，降低了PET/CT诊断恶性病变的敏感度。

## 6 GGNs的自然生长史

研究GGNs的自然生长史对于指导GGNs的随访、治疗具有重要意义。GGNs生长较实性结节缓慢^[33]^，且与多种因素有关^[34-36]^；体积倍增时间是反映结节自然生长状况的重要指标，关于GGNs的平均倍增时间目前仍不明确，但大量研究证实体积、密度的改变在结节随访中均有重要意义。在Matsuguma等^[37]^的研究中共纳入178例GGNs，其中pGGNs 98例，mGGNs 76例，中位随访时间为29（1-136）个月，定义直径增长2 mm及以上为结节生长；在pGGNs中，2年生长率及5年生长率分别为13%、23%；而在mGGNs中，分别为38%、55%。Kobayashi等^[38]^采用相同标准定义结节生长，对120例GGNs进行观察，中位随访时间为4.2年，共观察到34例（28.3%）结节生长；进一步分析发现：吸烟史、结节直径1.1 cm-3.0 cm（*vs*直径≤1 cm）是结节生长的独立危险因素。该结论与Lee等^[36]^研究一致：在136例pGGNs、77例mGGNs中，pGGNs≥10 mm及mGGNs≥8 mm均提示结节增长较快。此外，另一项纳入125例GGNs的研究亦证实：结节大小、肺癌病史是GGNs生长的独立危险因素^[39]^。上述研究均表明：GGNs实性成分比例、直径大小、肺癌病史等因素均与结节生长有关。

此外，Eguchi等^[40]^的研究证实结节CT值也可影响GGNs的生长。通过HRCT随访124例pGGNs患者，中位随访时间为57.0个月，其中64例（51.6%）直径增大，其平均CT值较直径不变的结节高[（-602.9±90.7）HU *vs*（-705.7±77.7）HU, *P* < 0.000, 1]；同时，CT值≥-670 HU的pGGNs更易出现直径增大（*P* < 0.000, 1）。

在GGNs中，pGGNs是生长速度最为缓慢的类型，即使随访2年未见体积改变，仍不能完全排除恶性病变的可能。Chang等^[41]^使用低剂量CT对122例pGGNs进行随访，中位随访时间为59个月，结果显示：仅12例pGGNs（9.8%）体积增大，其中位体积倍增时间为769天，病理诊断证实其中11例为早期肺癌。在另一项研究^[12]^中，共89例pGGNs患者纳入随访；其中仅8例患者结节直径增大，3例CT值升高，其中位生长时间为20.6个月。

## 7 GGNs的分子病理特征

随着生物靶向治疗的进展，越来越多的研究开始着眼于肺癌相关的驱动基因，加强其管理、监测变得极为迫切。肺腺癌中较为常见的驱动基因包括*EGFR*、*ALK*、*KRAS*、*ROS1*、*HER2*、*p53*等，其中*EGFR*、*ALK*在*GGNs*中的研究较多。

不同驱动基因阳性率在不同群体中差异较大。Kobayashi等的研究^[42]^纳入104例GGNs，其中75%（78/104）检出驱动基因，*EGFR*、*KRAS*、*ALK*、*HER2*阳性率分别为64%、4%、3%及4%。在另一项纳入35例患者、60例GGNs的研究中，Wu等^[43]^通过手术标本检测了*EGFR*、*HER2*、*KRAS*、*BRAF*、*EML4-ALK*、*ROS1*等基因，仅6例患者驱动基因改变情况一致。

既往研究发现驱动基因阳性率与腺癌分型、实性成分比例、结节直径等因素有关。EGFR阳性者多为MIA/IA，阴性者多为AAH/AIS^[42]^；且其21外显子缺失在贴壁状为主型IA中更为常见^[44]^。同时，Yang等^[45]^的研究证实EGFR突变与GGNs的直径及体积密切相关。但关于实性成分比例与驱动基因的关系，目前尚无一致结论。Takatoshi等^[46]^的研究发现EGFR突变率在pGGNs、mGGNs中分别为36%、45%，而*p53*突变在pGGNs（*n*=14）中均为阴性，在mGGNs（*n*=11）中存在6例（55%）阳性；这与Wang等^[47]^的结论一致，他们发现在212例Ia期肺癌患者中，实性成分比例越高，EGFR及KRAS突变率越高；而Yang等^[45]^则指出*EGFR*/*KRAS*突变、*EML4-ALK*融合在GGNs、实性结节中频率相当。该论点有待后续大样本量的高质量研究证实。

**1:**
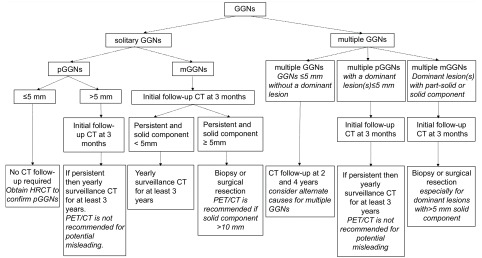
GGNs诊治流程建议 Suggested guideline for management of GGNs. CT: computed tomography; HRCT: high resolution CT; GGNs: ground-glass nodules; pGGNs: pure GGNs; PET/CT: positron emission tomography/computed tomography.

驱动基因的改变还与结节进展状况密切相关，既往多项研究均已证实*EGFR*突变与结节生长具有相关性。Kobayashi等的研究^[42]^发现EGFR阳性的GGNs在随访过程中体积有增长（*P* < 0.01），而阴性者结节体积无明显改变（*P* < 0.01）；这一结论与Takatoshi等^[46]^的研究一致。

## 8 GGNs诊治流程

美国Fleischner学会^[48]^综合IASLC、ATS及ERS的内容，对GGNs的诊治流程提出了建议。

GGNs为肺结节的一种特殊类型，其基因突变、生长代谢等均与其他肺结节不同。GGNs对于肺腺癌具有高度提示作用，合理应用HRCT及PET/CT等手段有助于早期发现肺癌，改善预后。
